# Optimization of Polyamide Pulp-Reinforced Silica Aerogel Composites for Thermal Protection Systems

**DOI:** 10.3390/polym12061278

**Published:** 2020-06-03

**Authors:** Mariana E. Ghica, Cláudio M.R. Almeida, Mariana Fonseca, António Portugal, Luísa Durães

**Affiliations:** University of Coimbra, CIEPQPF, Department of Chemical Engineering, 3030-790 Coimbra, Portugal; meghica@eq.uc.pt (M.E.G.); claudio@eq.uc.pt (C.M.R.A.); mariana.fonseca.64@gmail.com (M.F.); atp@eq.uc.pt (A.P.)

**Keywords:** silica-based aerogel, Kevlar^®^ pulp, reinforcement, thermal insulation

## Abstract

The present work describes for the first time the preparation of silica-based aerogel composites containing tetraethoxysilane (TEOS) and vinyltrimethoxysilane (VTMS) reinforced with Kevlar^®^ pulp. The developed system was extensively investigated, regarding its physical, morphological, thermal and mechanical features. The obtained bulk density values were satisfactory, down to 208 kg·m^−3^, and very good thermal properties were achieved—namely a thermal conductivity as low as 26 mW·m^−1^·K^−1^ (Hot Disk^®^) and thermal stability up to 550 °C. The introduction of VTMS offers a better dispersion of the polyamide fibers, as well as a higher hydrophobicity and thermal stability of the composites. The aerogels were also able to withstand five compression-decompression cycles without significant change of their size or microstructure. A design of experiment (DOE) was performed to assess the influence of different synthesis parameters, including silica co-precursors ratio, pulp amount and the solvent/Si molar ratio on the nanocomposite properties. The data obtained from the DOE allowed us to understand the significance of each parameter, offering reliable guidelines for the adjustment of the experimental procedure in order to achieve the optimum properties of the studied aerogel composites.

## 1. Introduction

Silica-based aerogels are lightweight amorphous materials, having very high pore volume (>90%) and usually a mesoporous network, with average pore diameter between 20 and 40 nm, which provide a very high specific surface area [1]. Another important property of silica aerogels is their very low thermal conductivity (0.015–0.025 W·m^−1^·K^−1^) [1,2], associated with non-flammable character, making them suitable materials for thermal insulation systems, even in extreme temperature environments [1]. Thus, this lightweight engineered material brings great improvement for thermal protection systems, especially in Space, but still needs to be optimized for Space conditions. However, it is important to note that NASA has already used this technology to capture Space dust particles and in Mars exploration vehicles; research has also been conducted to use these materials for thermal insulation of Space suits [1,2,3,4].

Despite the mentioned exceptional properties, silica aerogels possess fragile interparticle necks, which do not allow them to maintain the monolithic form under stress loads, limiting their use in more ambitious applications [1,4,5]. Regarding this, hybridization may be one of the solutions to overcome the mechanical brittleness of silica aerogels. This is promoted by co-condensation of organosilanes (e.g., methyltriethoxysilane (MTES), bis(trimethoxysilyl)hexane (BTMSH) and vinyltrimethoxyxilane (VTMS)) [6,7] as co-precursors in order to induce flexibility [8,9]. The use of polymers as a strategy for reinforcement of the silica aerogels structure has also been proven as a good technique for improving their mechanical properties; however, this procedure generally increases the aerogel density and thermal conductivity, and these polymers usually degrade at temperatures lower than 400 °C [5].

In addition to the referred reinforcement strategies, fibers can be incorporated into aerogels to increase their robustness [7,10]. The fiber web supports the aerogel and decreases the aerogel bulk size in the composite matrix, i.e., it becomes an aerogel consisting of small monolithic volumes filling the spaces of the fiber network. Some examples of fibers used for this reinforcement purpose include polymeric fibers, carbon nanofibers and glass fibers [5,7,10]; their addition leads to improvement of mechanical properties, while only slightly increasing bulk density and thermal conductivity [11,12]. The fibers can be added in different forms, for example original fibers, pre-processed fibers (e.g., cut or ground) or non-woven fiber felts [13], and should be introduced into the sol phase, so that the gel is already formed in their presence, resulting in a fiber-aerogel composite after drying.

Several factors have to be evaluated when incorporating fibers in aerogels, such as their dispersion and compatibility with the sol, hence the chemical composition of the fibers is an important characteristic to consider [5,13]. Li et al. reported that the highest potential for reinforcing the silica aerogel network is offered by the fibers with small diameter, high flexibility and low density; additionally, their compatibility with the sol increases if they are organic, such as aramid fibers [13]. However, the systems studied by Li et al. showed a limited thermal stability (285 °C; air atmosphere).

The best-known polyamide fibers are synthesized by DuPont™, with the commercial trade name Kevlar^®^ (para-aramid) and Nomex^®^ (meta-aramid). They are non-flammable and self-extinguishing materials, and present limiting oxygen index values around 30. Aramid type fibers have thermal stability up to 400 °C, in the case of meta-aramid, and 500 °C for the para-aramid configuration, and the corresponding thermal conductivity values are 250 and 300 mW·m^−1^·K^−1^, respectively. Meta aramids are, in general, more flexible (~30% elongation at break) when compared with para-aramids (~3%), which makes them more appropriate for applications involving shape adaptation [14,15].

Silica aerogels have already been prepared with the addition of different aramid structures (fibers, pulp, honeycombs) [13,16,17,18,19]. However, none of them presented an organically modified silica system based on tetraethoxysilane (TEOS) and vinyltrimethoxysilane (VTMS), which can bring new interaction features between the fibers and the matrix. According to our knowledge, no work has been yet performed for the development of an aramid-silica aerogel composite using the referred silica system and reinforced with Kevlar^®^ pulp.

Thus, the purpose of the present work was the development of Kevlar^®^ pulp-reinforced silica aerogel composites for thermal insulation at high temperature (at least up to 500 °C) without significant disintegration. Several composites were prepared by using different ratios of co-precursors, and the pulp amount and the ratio of the number of moles of solvent and precursors were also varied. A design of experiment (DOE) was carried out to investigate the influence of these parameters on the final material properties and predict the optimum experimental conditions for the preparation of an aerogel with low bulk density, thermal conductivity and good flexibility. The developed Kevlar^®^ pulp/silica aerogel composites were characterized by different techniques to assess their physical, morphological, thermal and mechanical properties.

## 2. Materials and Methods

### 2.1. Materials

Tetraethoxysilane (TEOS, 98% purity, Acros Organics, Geel, Belgium), vinyltrimethoxysilane (VTMS, 98% purity, Acros Organics, Geel, Belgium), ethanol (purity ≥ 99.8%, ThermoScientific, Waltham, MA, USA), ammonium hydroxide (25% NH_3_ in H_2_O, Fluka Analytical, Darmstadt, Germany), oxalic acid anhydrous (purity ≥ 99%, Fluka, Darmstadt, Germany), hexamethyldisilazane (HMDZ, 98.5% purity, ThermoScientific, Waltham, MA, USA), n-heptane (purity ≥ 99.5%, ThermoScientific, Waltham, MA, USA) and Kevlar^®^ pulp (KP, DuPont, Wilmington, USA, 0.5–1.0 mm length) were used as received. High purity water was used to prepare the solutions of oxalic acid (0.01 M) and ammonium hydroxide (1.0 M) catalysts.

### 2.2. Synthesis of the Aerogel Composites

TEOS and TEOS/VTMS gels were prepared using a two-step acid-base catalyzed sol-gel process at 27 °C. Firstly, the precursors were diluted in ethanol, followed by addition of oxalic acid catalyst for hydrolysis and the solution was then stirred for 30 min. After 18 h, KP was added and stirred for 30 min for homogeneous dispersion, followed by the addition of NH_4_OH solution. The ratios between the number of moles of solvent and Si (*S*) tested in this work were 6, 10 or 14. The Si:acid water:basic water molar ratio was kept constant at 1:4:4. The content of KP in different syntheses was between ca. 2% and 8% of the dried aerogel weight. The higher amount, ca. 6 mg·ml^−1^, was the maximum that could be dispersed in the sol and keep all the fibers incorporated.

TEOS/VTMS gels were prepared with the following molar ratio combinations of precursors: 1:0; 0.9:0.1; 0.8:0.2; 0.75:0.25; 0.7:0.3; 0.5:0.5. The corresponding formulations are designated as TEOS, TEOS_0.9_/VTMS_0.1_, TEOS_0.8_/VTMS_0.2_, TEOS_0.75_/VTMS_0.25_, TEOS_0.7_/VTMS_0.3_ and TEOS_0.5_/VTMS_0.5_, respectively.

The prepared gels were transferred to an oven for aging for 6 days at 27 °C, followed by washing with heptane during 48 h at 50 °C. In order to obtain a hydrophobic aerogel composite, a surface modification was performed with a mixture of HMDZ in n-heptane (15% v/v) during 16 h at 50 °C, followed by 3 h washing with n-heptane at 50 °C to remove the unreacted compounds. All samples were finally dried in the oven during 4 h at 60 °C, then 2 h at 150 °C to provide APD aerogel composites (Figure S1).

Regarding the samples’ nomenclature, extensions were added to the previous designations, for distinction of the different conditions, as follows: SM—surface modified; NSM—non surface modified; KP—Kevlar^®^ pulp added. When the referred extensions are not used for simplicity of the nomenclature, it means that the samples were reinforced with KP and surface modified.

### 2.3. Characterisation of the Composites

The chemical modification of the aerogels was assessed by FTIR (FT/IR 4200, Jasco, Easton, USA), collecting the spectra of KBr pellets with the samples (0.1–0.2% wt.), between a wavenumber of 4000 and 400 cm^−1^, with 128 scans and 4 cm^−1^ of resolution. The degree of hydrophobicity was determined through contact angle measurements, by an OCA 20 system (Dataphysics, Filderstadt, Germany), at room temperature, using the sessile drop method and high purity water.

For the bulk density (*ρ*_b_) determination, the weight and dimensions of regular samples were measured (with a microbalance of 10^−5^ g precision and a caliper of 0.01 mm resolution). The linear shrinkages were obtained by measuring the samples’ diameter before and after the solvent exchange and drying steps. In order to evaluate the aerogel skeletal density (*ρ*_s_), He pycnometry (Accupyc 1330, Micromeritics, Norcross, GA, USA) was used.

The specific surface area (*S*_BET_) was determined by nitrogen gas adsorption at 77 K (ASAP 2000, Micromeritics, Norcross, GA, USA) applying the Brunauer–Emmett–Teller (BET) theory in the relative pressure interval 0.05–0.3 of the adsorption isotherm.

The BET equation [20] can be arranged as:

*P*/[*V*_ads_ (*P*_0_ − *P*)] = 1/(*V*_m_*c*) + [(*c* − 1)/(*V*_m_*c*)] *(P/P*_0_*)* (1)

where *P* and *P*_0_ are the equilibrium and the saturation pressure of adsorbate at the temperature of adsorption, *V*_ads_ is the adsorbed gas volume and *V*_m_ is the monolayer adsorbed gas volume, and *c* is the BET constant. A plot of *P*/[*V*_ads_ (*P*_0_ − *P*)] against (*P*/*P*_0_) should give a linear profile, with intercept 1/(*V*_m_ *c*) and slope (c − 1)/(*V*_m_ *c*). After determining *V*_m_, *S*_BET_ can be calculated from:

*S*_BET_ = *a* [(*P*_0_*V*_m_)/(*R T*)] *N*_A_ (2)

where *a* is area of a N_2_ molecule (16.2Å^2^), *N*_A_ is the Avogadro number (6.023 × 10^23^ mol^−1^) and *T* is the standard temperature (273 K).

For the determination of the samples’ porosity, Equation (3) was used:

Porosity (%) = (1 − *ρ*_b_/*ρ*_s_) × 100 (3)

Pore volume (*V*p) and average pore diameter (*d*_pore_) could be also estimated considering Equations (4) and (5), usually applied for aerogels exhibiting a highly constrained network [21]:

*V*p (cm^3^ g^−1^) = 1/*ρ*_b_ − 1/*ρ*_s_ (4)

*d*_pore_ (nm) = 4 (*V*p)/*S*_BET_ (5)

The pore size distribution in the range of mesopores and sub-micrometric macropores was obtained by the desorption branch of the isotherms (N_2_ relative pressure from 1.0 to 0.5), using the Barrett, Joyner and Halenda model (BJH), which correlates the diameter of pores with the relative pressure by the Kelvin equation.

Scanning electron microscopy (SEM) was used to investigate the aerogel composites morphology and microstructures. The images were taken with a Compact/VP Compact FESEM (Zeiss Merlin, Carl Zeiss Microscopy GmbH, Jena, Germany), after coating the samples with a thin gold layer by Physical Vapour Deposition, for 60 s.

The thermal stability of selected aerogels was assessed using a Simultaneous Differential Scanning Calorimeter, combining thermogravimetric analysis and differential scanning calorimetry, DSC/TGA (SDT Q500, TA Instruments, New Castle, PA, USA), from room temperature to 800 °C, with a heating rate of 10 °C·min^−1^.

Thermal conductivity, *k*, was measured with a Thermal Constants Analyser TPS 2500 S (Hot Disk^®^,Gothenburg, Sweden ), using the transient plane source method with two similar samples with the sensor in between and maintained at 20 °C. In general, the measurements were performed with the sensor 5465 (diameter = 3.2 mm), due to limitations of samples size. For the most promising samples, with lowest densities and thus larger, the sensor 5501 could be used (diameter = 6.4 mm), providing more reliable values.

The mechanical properties were obtained by uniaxial compression-decompression tests in an Inspekt mini-series (Hegewald and Peschke, Nossen, Germany), with a speed of 1 mm·min^−1^ using two load cells: one of 50 N to evaluate the Young’s modulus of the samples from a strain of 0% to 15%, and another of 3 kN from a strain of 0% to 25% and recovery to assess the dimensional stability. For these tests, the samples were cut into small cubic pieces of approximately 1.0 cm^3^ and all tests were performed in duplicate. A 5-cycle compression-decompression test (up to 25% strain) was also performed for one of the best samples.

### 2.4. Design of Experiments (DOE) Methodology

For DOE analysis, three factors were chosen for the screening: % of co-precursor (% CoP), % wt. of KP (% KP) and EtOH/Si molar ratio (*S*). Initially, a full factorial (2^3^ runs) with two levels for each factor (the lowest and highest) and one central point was evaluated. The levels for the factors (*S* from 6 to 14, % KP from 5 to 8 and % CoP from 0 to 50) were established based on preliminary tests (Figure S2), and the bulk density (*ρ*_b_), thermal conductivity (*k*) and Young’s modulus (*Y*_M_) were chosen as responses for the DOE analysis. The influence of the main parameters, as well as their first order interactions, on the three responses (*ρ*_b_, *k* and *Y*_M_) were evaluated using the standard least squares fit to obtain the models.

Since the system has shown some complexity, an additional DOE trial was performed and a central composite design was planned for surface response analysis. Moreover, the global optimization of the system with two or more responses requires the establishment of a desirability function [22], which may support the selection of the best synthesis conditions for preparation of the aerogel composites. This was also performed at a later stage of the analysis.

The DOE analysis was implemented using the JMP statistical software from SAS.

## 3. Results and Discussion

The DOE was performed to study the influence of *S*, VTMS co-precursor amount and KP amount on three key properties, the bulk density (*ρ*_b_), thermal conductivity (*k*) and Young’s modulus (*Y*_M_), in order to identify guidelines for the synthesis conditions. The systems were also characterized by other techniques to obtain additional information about their chemical, structural-morphological, thermal and mechanical features. The prepared aerogels with different combinations of parameters are listed in Table 1, together with their main properties.

### 3.1. Design of Experiments (DOE)

**Figure 1:**
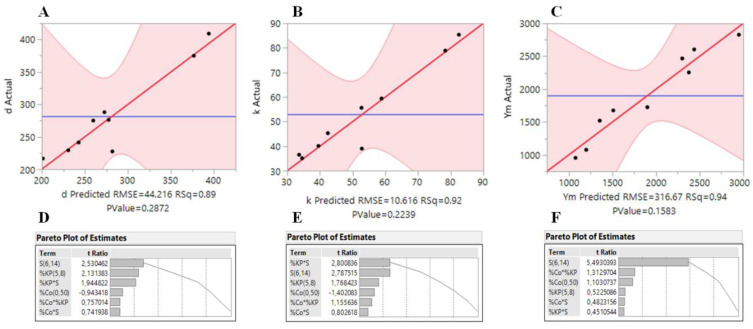
Actual vs. predicted responses (**A**–**C**) and Pareto plots (**D**–**F**) resulting from full factorial (2^3^). Bulk density (*d*) in kg·m^−3^, thermal conductivity (*k*) in mW·m^−1^·K^−1^ and Young’s modulus (*Y*m) in kPa.

In order to confirm the accuracy of the models, plots of the real responses (bulk density, thermal conductivity and Young’s modulus) of the samples versus the predicted responses are presented in Figure 1A–C. In general, the models fit well to the data, with *R*^2^ values of 0.89, 0.92 and 0.94 for *ρ*_b_, *k* and *Y*_M_, respectively. Pareto plots of the estimates obtained for the responses are also shown in Figure 1D–F, where it can be seen that the *S* factor has the highest influence in the responses. However, all the factors appear to have a non-negligible effect in the obtained responses, which makes the system much more complex.

**Figure 2:**
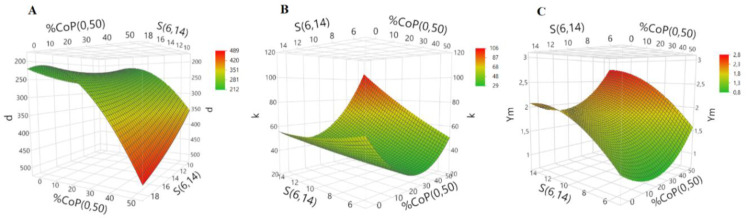
Surface responses for (**A**) bulk density (*d*, kg m^−3^), (**B**) thermal conductivity (*k*, mW·m^−1^·K^−1^) and (**C**) Young’s modulus (*Y*m, MPa).

For a deeper understanding of the system, surface responses were obtained using a central composite test design model. The surface responses for bulk density, thermal conductivity and Young’s modulus are represented in Figure 2, but taking only the most influent factors.

The factors that show major influence on the bulk density are % CoP and *S*, in agreement with previous studies [23,24]. The surface response indicates that the lowest values for bulk density are obtained for the following most favorable conditions: % CoP = 20, % KP = 8 and *S* = 6.

A similar trend was observed for thermal conductivity, which is also mostly influenced by the % CoP and *S*. The response surface graph of thermal conductivity showed that for the lowest value of *k*, the optimum values for % CoP, % KP and *S* are 24, 8 and 6, respectively.

Regarding the mechanical behavior, the most dominant factor for the *Y*_M_ is *S*. In fact, the model indicates that, with an increase in *S* value, the *Y*_M_ also increases. The lowest value was achieved using % CoP = 19, % KP = 6.5 and *S* = 6.

Therefore, an optimum aerogel composite is likely to be obtained in the following range of conditions: % CoP = 19–24, % KP = 6.5–8 and *S* = 6. In fact, the results in Table 1 are in good agreement with this trend.

The desirability function method, already used for aerogel samples [21], was applied here in order to obtain the global minimum value for density, thermal conductivity and Young’s modulus simultaneously, and the weights of 0.4, 0.4 and 0.2 for these responses were adopted, respectively (giving less significance to the mechanical properties). Under these circumstances, the optimum preparation conditions were found to be % CoP = 18, % KP = 7.2 and *S* = 6, corresponding to the following optimum response values: *ρ*_b_ = 0.173 g·cm^−3^, *k* = 27.6 mW·m^−1^·K^−1^ and *Y*_M_ = 1.1 MPa.

### 3.2. Chemical Characterisation

**Figure 3:**
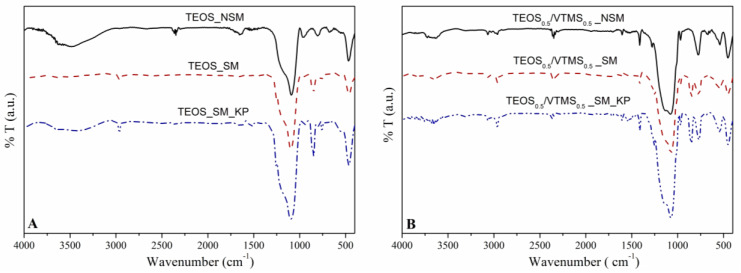
FTIR spectra before (NSM) and after surface modification (SM), and after adding fibers (KP), for (**A**) TEOS and (**B**) TEOS_0.5_/VTMS_0.5_ samples.

**FTIR analysis**. This analysis allowed to observe the constituents of the network, as well as to evaluate the success of surface modification. For all spectra (Figure 3), the strong Si–O–Si vibration bands near 460 cm^−1^ (deformation), 788 cm^−1^ (symmetric stretching) and 1084 cm^−1^ (asymmetric stretching) confirm the SiO_2_ network [25]. In Figure 3B, the presence of VTMS was confirmed by the appearance of the following bands: (i) at 540 cm^−1^, due to the twisting vibration of H derived from vinyl groups, (ii) at 1412 cm^−1^, which corresponds to the deformation vibration of =C–H groups, (iii) at 1605 cm^−1^ from the stretching vibration of C=C, and (iv) between 3000 to 3070 cm^−1^, attributed to the stretching of =C–H groups [7,25].

After surface modification with HMDZ, the bands related to –OH groups became weaker, indicating an increase in the hydrophobic character of the nanocomposites. It is also perceptible a higher intensity of the bands at 2800–3000 cm^−1^ and at ca. 1250–1280 cm^−1^ (stretching and deformation vibrations of –C–H groups, respectively) and the appearance of the absorption band at 848 cm^−1^, which results from the stretching of the Si−CH_3_ bonds. This indicates that the aerogel nanocomposites have been efficiently modified.

Three main bands are observed due to the presence of KP at: ca. 3330 cm^−1^, which is assigned as the –NH– stretching vibration; 1650 cm^−1^, representing the stretching of –C=O bonds; and 1542 cm^−1^, which is characteristic of the –NH– bending [26].

**Contact angle measurements**. To evaluate the wettability of the prepared composites, the water contact angles (WCA) were measured for samples in different conditions. The obtained WCA are reported in Table 1. It is well known that the hydrophobicity of a sample is strongly dependent on the chemical properties of the surface [27,28]. Since the samples were subjected to a surface modification step with a silylation agent (HMDZ), the modified materials are hydrophobic or even superhydrophobic in some cases, with contact angles ranging from 137° to 156°. After silylation, the hydroxyl groups were replaced by methyl groups in the silica surface, which leads to an increase in the hydrophobic character, by reducing the surface free energy. The surface modification was also confirmed by FTIR analysis (Figure 3), as already discussed. The hydrophobic character ensures a high durability of the materials when subjected to atmospheric moisture [1].

### 3.3. Structural and Morphological Features

**Figure 4:**
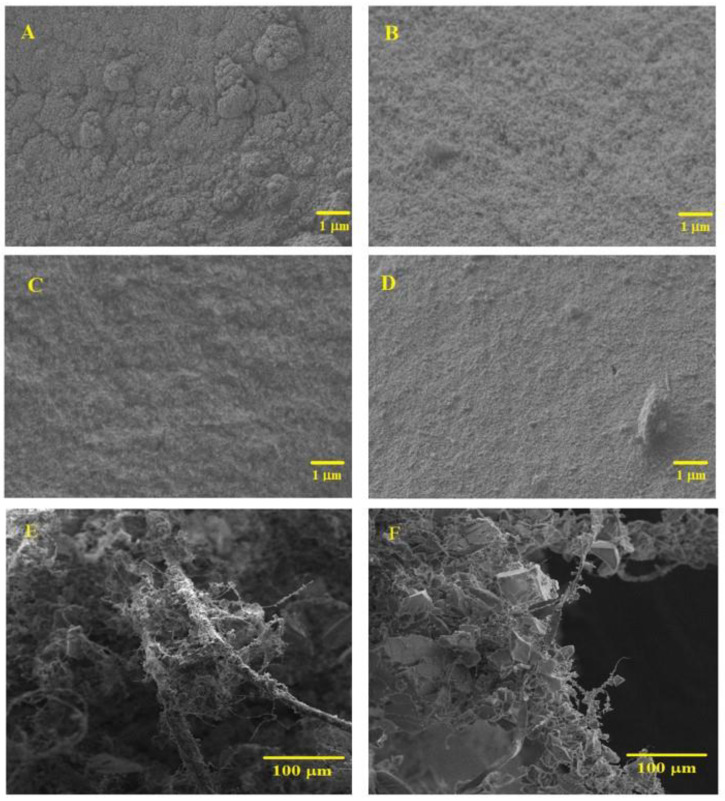
SEM images of aerogel composites obtained from TEOS (*S* = 10) (**A**) before and (**B**) after silylation. Same magnification images for silylated samples obtained from (**C**) TEOS (*S* = 6) and (**D**) TEOS_0.7_/VTMS_0.3_ (*S* = 10). Fiber dispersion in the TEOS-derived matrix with (**E**) *S* = 6 and (**F**) *S* = 14.

**SEM analysis**. The morphology and microstructure of the aerogel nanocomposites were analyzed by SEM. The micrographs of different configurations are shown in Figure 4. All samples exhibited an interconnected three-dimensional matrix (Figure 4A–D). In Figure 4A,B, a comparison between TEOS-based aerogel (*S* = 10) before and after surface modification is presented. After silylation (Figure 4B), the structure is more porous and less agglomerates were formed, due to the Si–(CH_3_)_3_ groups attached on the surface of the nanocomposites. By decreasing the *S* value (*S* = 6, Figure 4C), a less porous network was obtained, as expected. The introduction of VTMS as a co-precursor in the matrix (Figure 4D) led to a smooth and regular network, revealing less heterogeneity of the medium during gel formation, which may be due to a higher affinity of this matrix to KP fibers, compared to the system only with TEOS. Thus, the type of precursor, the solvent/Si molar ratio (*S*) and surface modification have a significant influence on the microstructure of the samples.

In Figure 4E,F, it is possible to observe that KP fibers form a reinforcing network that is covered by the silica matrix, in this way providing monolithicity to the aerogels. In samples prepared with lower *S* value (Figure 4F), a better coverage of the fibers by the silica phase is observed, as expected.

**Linear shrinkage**. Shrinkage of the samples occurred both in diameter and height, since the pulp in the matrix has a random orientation. The diameter of the samples was measured after ageing/washing/surface modification and after drying. The total shrinkage values varied between 14.4% and 39.7% (Table 1). However, in general, main part of shrinkage (10%–20%) was observed after solvent exchange and surface modification steps (Table S1). This happens due to a swelling/de-swelling phenomenon that KP suffers during the synthesis protocol of aerogels. First, when dispersed in ethanol, the KP swells. After solvent exchange, when ethanol is replaced by heptane, the phenomenon reverses. The swelling occurs because ethanol may easily form hydrogen bonds with amine groups of the Kevlar^®^ pulp and the apolar tail of ethanol increases the space between the fibers and decreases their interconnection. During the washing step, ethanol is removed and exchanged by heptane, a non-polar solvent that is not able to maintain the hydrogen bridges. Thus, de-swelling of pulp occurs, leading to a decrease in the gel volume.

The linear shrinkage after drying decreased with the increase in KP amount from 5 to 8%wt., in the case of TEOS-based aerogels. With the introduction of VTMS, the shrinkage is almost independent of KP amount. In all cases, the linear shrinkage increases with increase in the *S* value, as well as with increase in the VTMS content (Table 1). This may be explained by the less interconnected silica network with higher *S*, which results in lower strength of the gel to withstand the drying stresses. The effect of VTMS in the shrinkage may also be explained by the less connectivity of the network with the increase in VTMS/TEOS ratio, since VTMS has one non-hydrolysable group.

**Bulk density**. The influence of the Kevlar^®^ pulp concentration, *S* ratio and the ratio of co-precursors on the bulk density was investigated, as mentioned in the DOE, being the results presented in Table 1. Generally, a lower bulk density was obtained for the systems with *S* = 6, reaching a value of 0.208 g·m^−3^ (the lowest) for the TEOS_0.75_/VTMS_0.25_ aerogel with 6.5% KP. By increasing the *S* from 6 to 10, *ρ*_b_ slightly increases in the case of the aerogels containing only TEOS and those with smaller amount of VTMS (TEOS_0.75_/VTMS_0.25_). A further increase in *S* led to additional increase in bulk density values for all systems. Moreover, by introduction of a higher amount of VTMS in the aerogel matrix (TEOS_0.5_/VTMS_0.5_), an increase in the bulk density value was observed. The highest bulk density value was obtained for the TEOS_0.5_/VTMS_0.5_ system with *S* = 14.

Hence, it can be concluded that the increase in KP concentration leads to a decrease in the bulk density, certainly due to the higher support provided by the fibers. On the other hand, it was observed that the increase in *S* value, as well as the increase in VTMS amount, led to higher bulk density values, in agreement with shrinkage results.

**Skeletal density, specific surface area and porosity**. These properties were studied for the samples used for the initial DOE analysis, but only for KP = 8% (due to lower bulk density observed).

The obtained values of the skeletal density of the selected aerogel samples are shown in Table 2; they varied between 1.13 and 1.48 g·cm^−3^ and are in agreement with those in literature for similar organically-modified aerogel systems [7]. The porosities, obtained from Equation (3), are in the interval 81%–84.5%, with only one exception of a sample with 2-fold higher bulk density (Table 2; sample TEOS_0.5_/VTMS_0.5_, *S* = 14 and KP = 8%).

**Figure 5:**
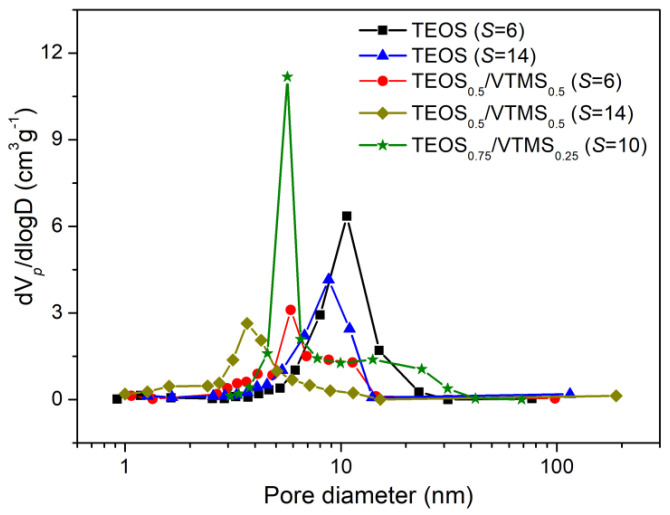
Pore size distribution in the region of mesopores and small macropores for different aerogel systems.

The specific surface areas and the pore size distributions of the prepared aerogels were evaluated from the nitrogen adsorption/desorption isotherms (Figure S3), and their values are presented in Table 2 and Figure 5. Notice that the average pore sizes in Table 2 were obtained from Equation (5) and not from N_2_ adsorption/desorption, in order to include all pore sizes.

As observed from Figure S3, the shape of the adsorption/desorption isotherms followed the type IV of IUPAC classification, characteristic of mesoporous materials, recognized by their hysteresis loop. Adsorption/desorption hysteresis shape of the aerogels corresponds approximately to H2 type of pores, indicating bottleneck-like pores. This is more evident in the samples with higher pore volume. The H2 type of pores is typical of disordered porous networks, with irregular pore shapes and complex interconnectivity.

The specific surface areas of the TEOS-based aerogels (~450 m^2^·g^−1^) were lower than those of TEOS-based aerogels synthesized in methanol [29], or synthesized in ethanol but that were silylated with TMCS [13,17,18,30], which is due to the more favorable synthesis compounds in the latter. However, these compounds (methanol and TMCS) represent more hazard for the operators and environment. The introduction of VTMS as a co-precursor leads to a slight increase in the surface area for *S* = 6 (Table 2), and for *S* = 14 the values are much higher (~615 m^2^·g^−1^), comparable with those for a similar TEOS/VTMS system [7]. The increase in surface area for the TEOS/VTMS-based aerogels may be explained by the higher amount of mesopores, since when vinyl groups were introduced in the aerogels, a reduction in the average pore diameter was observed (Table 2, Figure 5), probably due to more interaction between the KP and the organically modified matrix and to the higher shrinkage already discussed.

### 3.4. Thermal Characterisation

**Thermal conductivity**. The evolution of the thermal conductivity is in agreement with the trend of bulk density (Table 1)—generally both values decrease when using higher aramid pulp amount and lower *S* value. By increasing the *S* to 10, for the same KP amount, 6.5%, it is clear that the introduction of a small amount of VTMS in the aerogel matrix led to a decrease in 13.8% of the thermal conductivity, probably due to the reduction in pore size; a further increase in VTMS content contributes to almost double the *k* value for the TEOS_0.5_/VTMS_0.5_ sample, justified by an obvious dominant effect of the increase in bulk density.

The larger samples were measured with a sensor with higher radius, and the obtained thermal conductivities are significantly lower than the ones obtained by the smaller sensor (Table 1). The results of the larger sensor (5501) are more representative of the thermal conductivity of these samples, since they are assessed considering a larger volume for heat transfer. It is expected that for samples even larger, with size compatible with Guarded Hot Plate measurements (negligible boundary effect), the thermal conductivity may decrease further. Future work of scale-up of these aerogels will be performed to confirm this hypothesis.

**Figure 6:**
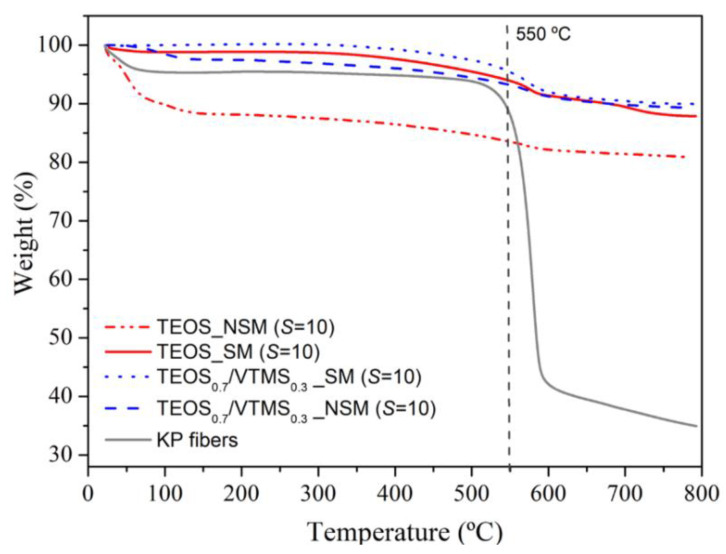
Thermograms for TEOS and TEOS_0.7_/VTMS_0.3_ samples containing 5%wt. of KP, with surface modification (SM) and non-surface modified (NSM).

**Thermal stability**. In order to assess the thermal stability of the KP-reinforced silica aerogel composites, a thermogravimetric analysis from room temperature to 800 °C was carried out under nitrogen atmosphere (Figure 6; Table S2). The first weight loss (~9%) of the non-silylated sample starts at room temperature and is due to adsorbed water and residual solvents/by-products (*T*_onset_ = 23.0 °C). It is noteworthy that in the case of samples containing VTMS this loss is much smaller (~2.3%) due to the greater hydrophobicity provided by the vinyl groups, even without silylation. The second mass loss is observed from ca. 350 up to 700 °C (*T*_onset_ = 450 °C), in an earlier stage attributed to the loss of structural hydroxyl groups and, in a later stage, to the thermal decomposition of the vinyl groups derived from VTMS co-precursor, decomposition of methyl groups attached to the silica surface after silylation, and decomposition of KP. After this phenomenon, it is expected the loss of the hydrophobic nature of the modified aerogel, as a result of the decomposition of methyl/vinyl groups. The final sample mass percentage was high (80%–90%) and is attributed to carbon and silica that remain in the crucible. In general, the prepared aerogel composites can be used at high temperature, up to 450–550 °C, for considerable periods of time, without significant thermal degradation and maintaining their size/monolithicity (Figure S4). In the case of TEOS/VTMS samples with surface modification, the thermal stability is higher, up to 550 °C (Table S2).

### 3.5. Mechanical Characterisation

**Figure 7:**
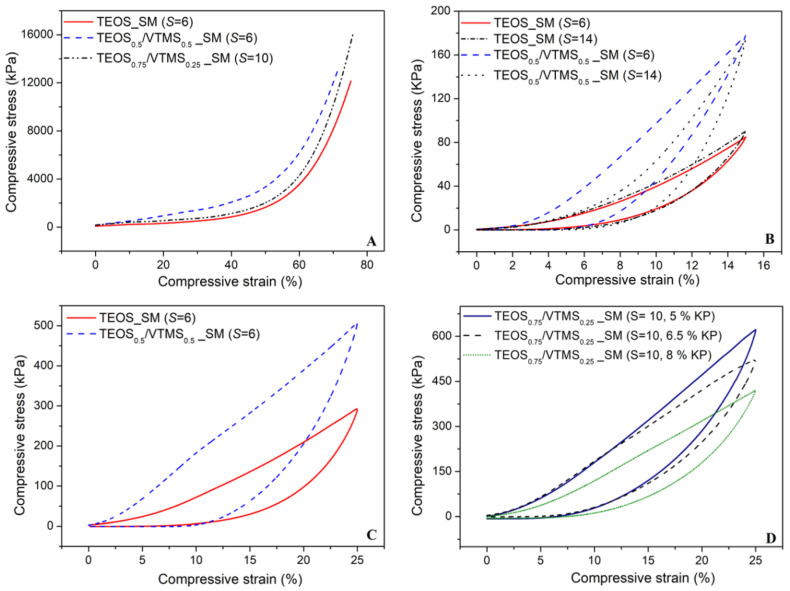
Stress–strain curves obtained by uniaxial compression with a load cell of 3 kN (**A**,**C**,**D**) and 50 N cell (**B**). (**A**) Silica aerogel composites (TEOS; TEOS_0.5_/VTMS_0.5_ and TEOS_0.75_/VTMS_0.25_; *S* = 6, 6 and 10) tested up to maximum densification. Effects of (**B**) *S* in TEOS and TEOS/VTMS-derived systems, (**C**) co-precursor and (**D**) different fibers content, under a uniaxial compression-decompression cycle.

The mechanical behavior of the silica aerogel composites reinforced with KP was assessed by uniaxial compression-decompression tests, consisting in loading and unloading of the samples with strain between 0% and 15% with a load cell of 50 N and between 0% and 25% with a load cell of 3 kN. A destructive test was also performed with the cell of 3 kN, up to the maximum allowed force in order to describe the mechanical behavior of the samples. The results of compressive stress versus strain obtained from the latter referred test is illustrated in Figure 7A for different samples. The curves can be divided essentially in three regions: linear stage, yielding stage and densification stage (elastic–plastic stage). In the beginning of linear stage (until ~6%), the compression curve slope is almost unchanged and the main bearing support during this phase corresponds to the open pores and the elastic bending of the KP fibers and pore walls. During the yielding stage (6%–40% strain), an increase in the stress is observed. The fibers in the aerogel matrix play the role of supporting skeleton, dispersing and transferring the external force to the whole sample and avoiding the collapse of the structure. This increase can also result from the elastic buckling of the pore walls [7,17]. In the final stage (>50% strain), the slope of the stress–strain curve increases significantly, due to the densification of the porous structure and correspondent increase in stiffness of the compressed specimen. Despite there being no expected chemical bonds between fibers and the silica matrix, the large interfacial adhesion increases the van der Waals’ force between the two phases [13]. The dispersion of KP fibers in the silica matrix is multidirectional and uniform, thus the external stress is transferred to the whole composite, avoiding stress concentration and being able to stand a larger external stress. As a consequence, the KP-reinforced aerogel composites can support higher compressive stresses when compared with composites reinforced with larger fibers [17].

The Young’s modulus was evaluated from the yielding stress–strain region and the values are presented in Table 1. Generally, the *Y*_M_ value increased with increase in *S* value, thus more flexible aerogel composites can be achieved at lower *S* values, which is in agreement with the bulk density decrease. In addition, the increase in VTMS amount in the matrix led to more rigid aerogel samples. Beside the effect of bulk density, this trend comes also probably from the interaction between vinyl group of the silica network with the aromatic ring of aramid via their pi-systems [31]. Regarding the fibers content, the influence on *Y*_M_ is not clear, although it has a tendency to decrease with the increase in the wt. % of KP, related to the decrease in bulk density.

The mechanical properties of the obtained aerogels are important to select the most suitable material for large volume applications. For example, good elasticity is required in Space applications (e.g., suits, pipes and panels) for adaptation to large bending. For this reason, the capacity of these materials to recover to their original shapes during unloading is very important. Thus, recovery tests were performed (Figure 7C,D) and the results indicate that in general, after 25% strain, the samples restore up to 77% their shape.

**Figure 8:**
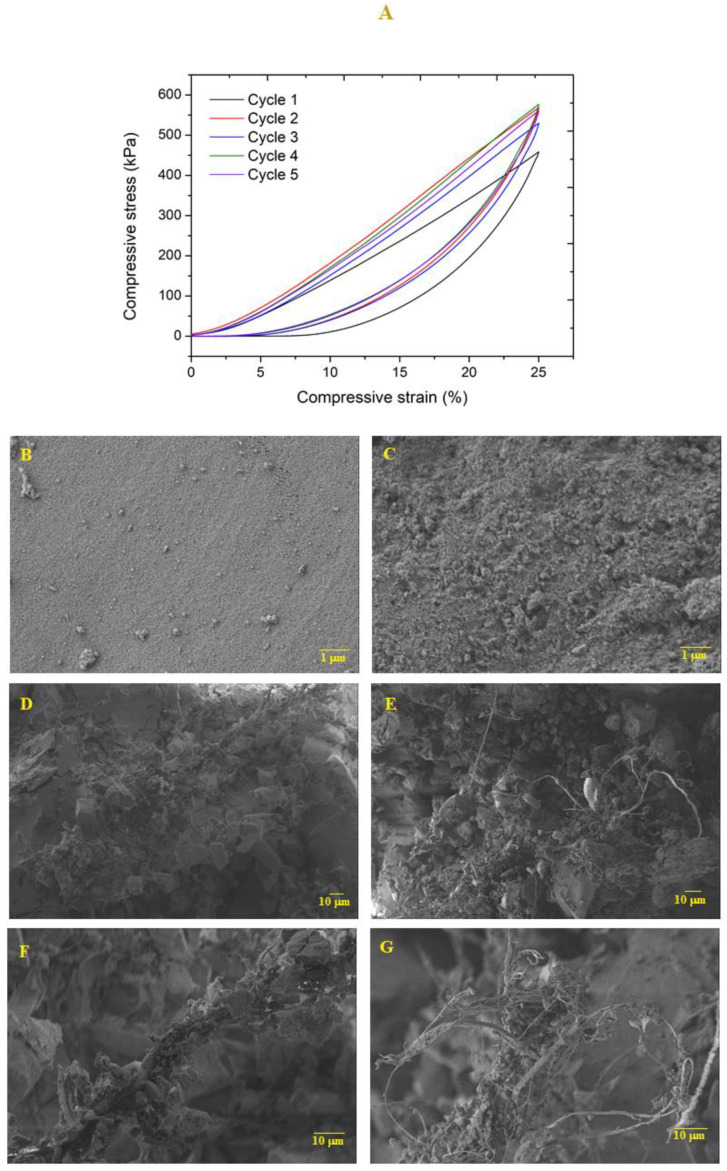
(**A**) Cyclic compressive test for TEOS_0.75_/VTMS_0.25_ (*S* = 10; KP = 6.5%). SEM images of (**B**) surface and (**D**,**F**) cross section before the compression test. SEM images of (**C**) surface and (**E**,**G**) cross section after 5 cycles of compression-decompression.

In order to evaluate the capacity of the nanocomposites to withstand dimensional loads, axial cyclic compression tests (5 cycles) were performed until 25% deformation, at a speed compression of 1 mm/min (Figure 8). After each cycle, the sample height was measured to evaluate its recovery capacity (Table S3). It featured a small decrease in height during the successive cycles. Regarding the maximum compressive stress, this value increased from ca. 450 to 550 kPa in the second cycle and remained almost unchanged during the next compressive cycles (Table S3). The sample retained its overall shape without noticeable fissures.

Sample microstructure was analyzed through SEM images (Figure 8B–G) before and after the cyclic compression-decompression. Surface images shows that, after the compression test, the sample topography became more irregular. By analyzing the cross section, it is possible to verify that after the compressive loads, the sample is more compact and the fibers are apparently less coated by the sol-gel matrix, resulting from the effect of axial compression forces. However, the integrity of the sample was maintained at the microstructural level after five compressive cycles, indicating that these nanocomposites are capable of supporting reasonable loads without fragmentation.

## 4. Conclusions

Silica aerogel composites were successfully prepared through APD using the co-precursor method and reinforcing the aerogel matrix with aramid pulp. After surface modification, the samples became hydrophobic and a significant decrease in bulk density was observed. The microstructure analysis of the composites showed that the fibers were covered with the silica matrix, acting as a supporting skeleton. The introduction of VTMS led to a smaller pore size and a more regular silica network, which indicate a good affinity with aramid fibers.

DOE analysis was performed to predict the influence of synthesis parameters on the material properties. The Pareto plots showed that the parameter with less influence was %wt. KP; in fact, the surface response analysis exhibited a higher dependence of the bulk density, thermal conductivity and Young’s modulus on % CoP and *S*. By using the desirability function with weighting factors, the optimized configuration was obtained when ~18% of CoP, 7.2%wt. KP and *S* of 6 were used.

Bulk density and thermal conductivity were favored when a higher amount of KP was used, as well as lower *S* and lower % CoP. Mechanical tests indicated that the samples present a lower *Y*_M_ for the lowest *S* values, and after the introduction of VTMS the composites become more rigid. Moreover, the best nanocomposites are able to withstand cyclic loads (with strain up to 25%) without significant change of their shape and microstructure. TG-DSC analysis demonstrated a high thermal stability of composites, up to 500–550 °C.

This combination of excellent properties indicates that the obtained aerogel composites have great potential to be used in the thermal insulation field, especially in high-temperature environments.

## Figures and Tables

**Table 1:** Properties of KP-reinforced silica aerogel composites developed in this work.

System	*S*	KP (% wt.)	Linear Shrinkage after Drying (%)	*ρ*_b_ (g/cm^3^)	*k* (mW/m·K)	*Y*_M_ (MPa)	Water Contact Angle (°)
TEOS	6	5.0	22.2	0.276 (±0.007)	59.4	1.52	141 ± 10
		8.0	16.5	0.216 (±0.004)	36.6 (32.2 ^a^)	1.08	138 ± 6
	10	6.5	22.1	0.211 (±0.011)	44.9	1.05	-
	14	5.0	35.1	0.288 (±0.001)	55.6	2.25	146 ± 6
		8.0	30.9	0.241 (±0.002)	45.3	2.60	152 ± 7
TEOS_0.75_/VTMS_0.25_	6	6.5	14.4	0.208 (±0.014)	34.7	1.71	-
	10	5.0	25.0	0.242 (±0.000)	31.3	1.81	
		6.5	22.5	0.214 (±0.008)	38.7 (28.7 ^a^)	1.81	156 ± 8
		8.0	23.4	0.231 (±0.011)	35.8	1.56	
	14	6.5	29.5	0.236 (±0.000)	46.0	1.24	-
TEOS_0.5_/VTMS_0.5_	6	5.0	20.6	0.275 (±0.005)	40.1	0.96	153 ± 6
		8.0	20.8	0.229 (±0.005)	35.1 (26.6 ^a^)	1.68	145 ± 4
	10	6.5	33.3	0.382 (±0.002)	70.5	2.99	-
	14	5.0	39.0	0.375 (±0.002)	78.9	2.47	137 ± 5
		8.0	39.7	0.409 (±0.005)	85.4	2.83	142 ± 6

^a^ These values were measured with the sensor 5501.

**Table 2:** Properties of KP-reinforced silica aerogel composites obtained by He pycnometry and nitrogen gas adsorption.

System	*S*	KP (wt.%)	*ρ*_s_ (g/cm^3^)	Porosity (%)	*S*_BET_ (m^2^/g)	*d*_pore_ (nm)
TEOS	6	8.0	1.175 (±0.079)	81.6	466.2 (±6.4)	32.4
	14	8.0	1.269 (±0.028)	81.0	428.4 (±5.9)	31.3
TEOS_0.75_/VTMS_0.25_	10	6.5	1.134 (±0.071)	81.1	619.9 (±5.9)	24.4
TEOS_0.5_/VTMS_0.5_	6	8.0	1.477 (±0.031)	84.5	488.6 (±4.4)	30.0
	14	8.0	1.253 (±0.019)	67.4	610.7 (±4.8)	10.8

## Supplementary Materials

The following data are available online at https://www.mdpi.com/2073-4360/12/6/1278/s1, Figure S1: Aerogel composites based on (A) TEOS, (B) TEOS_0.5_/VTMS_0.5_ and (C) TEOS_0.75_/VTMS_0.25_ precursor systems, with surface modification and *S* of (A,B) 6 and (C) 10; Figure S2: (A) The influence of KP content on the bulk density of the aerogels based on TEOS (□) without and (■) with surface modification (NSM and SM respectively), *S* = 10. (B) The influence of KP content on the bulk density of the aerogels based on TEOS_0.7_/VTMS_0.3_ (□) without and (■) with surface modification, *S* = 10. (C) The effect of co-precursors molar ratio on the bulk density of TEOS/VTMS aerogels with 5%wt. KP and (□) without surface modification or (■) with surface modification, *S* = 10. (D) The effect of *S* on the bulk density of aerogels of (■) TEOS and (▲) TEOS_0.7_/VTMS_0.3_ systems with surface modification; Figure S3: N_2_ adsorption (■) and desorption (■) isotherms for KP-reinforced silica aerogels; Figure S4: Aerogel composites based on TEOS_0.75_/VTMS_0.25_, with surface modification and *S* = 10, before (left) and after (right) a heat treatment test at 500 °C for 30 min. Table S1: Shrinkage during processing steps of KP-reinforced silica aerogel composites; Table S2: Thermogravimetric analysis data of KP-reinforced silica aerogel composites; Table S3: Recovery of sample height and maximum compressive stress after each compressive cycle.
