# Supplementary material for: Optimization of Polyamide Pulp-Reinforced Silica Aerogel Composites for Thermal Protection Systems
Source: Polymers (Basel). 2020 Jun 3;12(6):1278. doi: 10.3390/polym12061278 (PMC7361946; doi:10.3390/polym12061278)
Supplement: Supplementary file 1 [file polymers-12-01278-s001.pdf]

Supplementary Material

Optimization of polyamide pulp-reinforced silica aerogel composites for thermal protection systems

Mariana E. Ghica, Cláudio M. R. Almeida, Mariana Fonseca, António Portugal, Luísa Durães*

University of Coimbra, CIEPQPF, Department of Chemical Engineering, 3030-790, Coimbra, Portugal

***Corresponding author:** luisa@eq.uc.pt

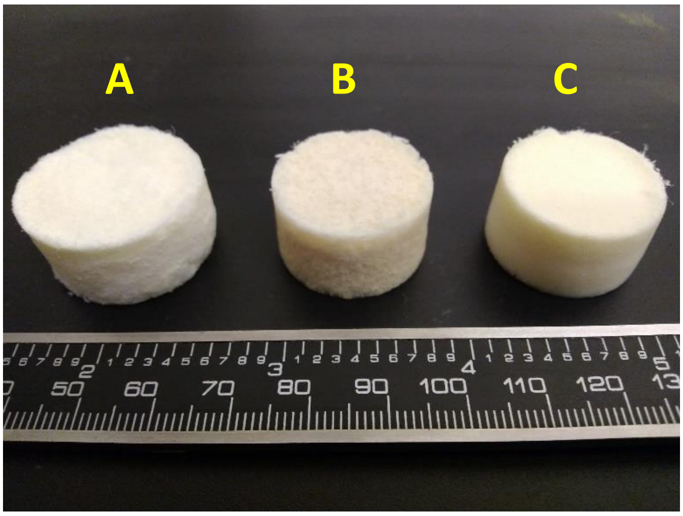

Figure S1: Aerogel composites based on (A) TEOS, (B) $TEOS_{0.5}/VTMS_{0.5}$ and (C) $TEOS_{0.75}/VTMS_{0.25}$ precursor systems, with surface modification and *S* of (A,B) 6 and (C) 10.

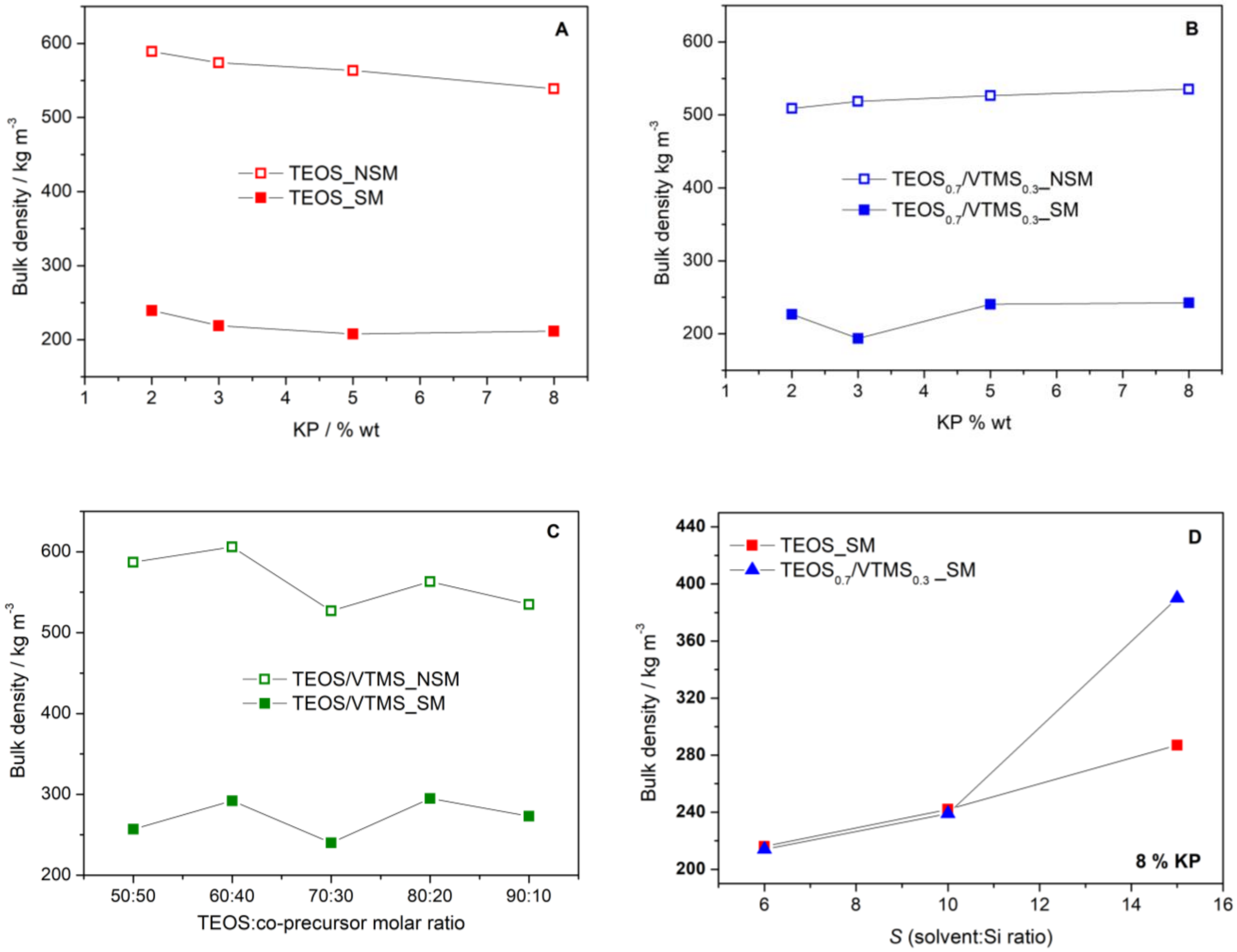

Figure S2: (A) The influence of KP content on the bulk density of the aerogels based on TEOS (□) without and (■) with surface modification (NSM and SM respectively), *S*=10. (B) The influence of KP content on the bulk density of the aerogels based on $TEOS_{0.7}/VTMS_{0.3}$ (□) without and (■) with surface modification, *S*=10. (C) The effect of co-precursors molar ratio on the bulk density of TEOS/VTMS aerogels with 5 %wt. KP and (□) without surface modification or (■) with surface modification, *S*=10. (D) The effect of *S* on the bulk density of aerogels of (■) TEOS and (▲) $TEOS_{0.7}/VTMS_{0.3}$ systems with surface modification.

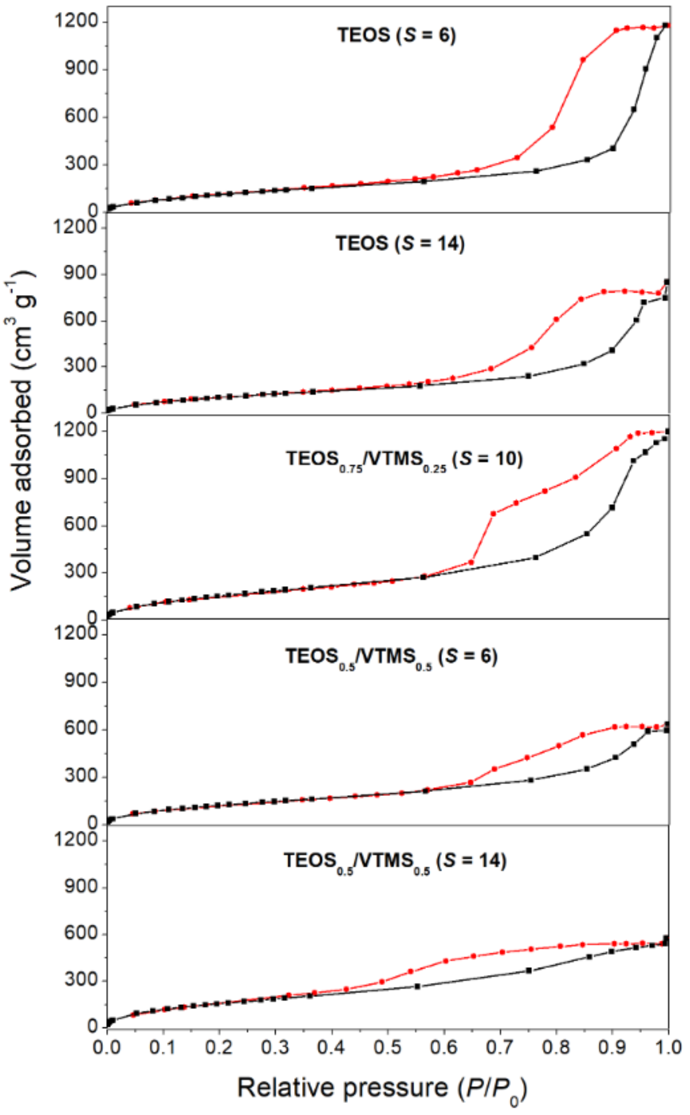

Figure S3: N_2 adsorption (■) and desorption (■) isotherms for KP-reinforced silica aerogels.

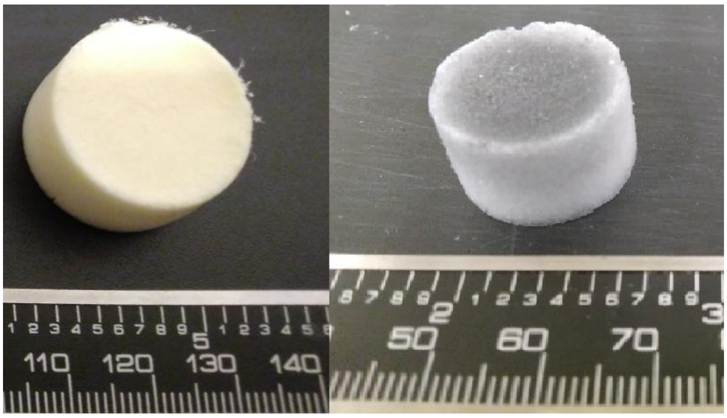

Figure S4: Aerogel composites based on $TEOS_{0.75}/VTMS_{0.25}$, with surface modification and *S*=10, before (left) and after (right) a thermal treatment test at 500 °C for 30 min.

Table S1. Shrinkage during processing steps of KP-reinforced silica aerogel composites.

System	*S*	KP (% wt.)	Linear shrinkage (after solvent exchange & surface modification) (%)	Total linear shrinkage (after drying) (%)
TEOS	6	5.0	13.2	22.2
	10	6.5	14.2	22.1
	14	5.0	25.2	35.1
$TEOS_{0.75}/VTMS_{0.25}$	6	6.5	7.6	14.4
	10	5.0	19.9	25.0
		6.5	17.5	22.5
		8.0	16.2	23.4
	14	6.5	20.6	29.5
$TEOS_{0.5}/VTMS_{0.5}$	6	5.0	9.6	20.6
	10	6.5	10.0	33.3
	14	5.0	27.1	39.0

Table S2: Thermogravimetric analysis data of KP-reinforced silica aerogel composites.

System	T_{onset} (°C)	T_{end} (°C)	Weight loss (%)	Phenomena	Weight loss at 500 °C (%)	Residue (%)
**TEOS *S*10; KP 5 %wt. NSM**	23.0	66.5	9.0	Removal of EtOH/heptane	4.8	80.8
	103.5	110.4	1.9	Removal of H_2O		
	456.1	588.8	5.8	OH groups/KP decomposition		
**$TEOS_{0.7}/VTMS_{0.3}$ *S*10; KP 5 %wt. NSM**	63.1	121.4	2.3	Removal of EtOH/H_2O	7.5	84.3
	461.9	616.9	7.7	OH groups/KP/C=C groups decomposition		
**TEOS *S*10; KP 5 %wt. SM**	44.3	66.7	0.5	Removal of EtOH/heptane	11	88.0
	492.6	587.5	7.8	-CH_3 groups/KP decomposition		
	690.3	740.9	3.3	Second phase degradation of -CH_3 groups Removal of SiO_2 defects		
**$TEOS_{0.7}/VTMS_{0.3}$ *S*10; KP 5 %wt. SM**	495.3	598.5	9.6	-CH_3 groups/KP/C=C groups decomposition	10.2	90.0

Table S3: Recovery of sample height and maximum compressive stress after each compressive cycle.

**Compressive cycle**	**Recovery (%)**	**Maximum compressive stress (kPa)**
1º	95 %	460
2º	93 %	570
3º	92 %	530
4º	89 %	575
5º	88 %	560
